# Pyridyl thiosemicarbazide: synthesis, crystal structure, DFT/B3LYP, molecular docking studies and its biological investigations

**DOI:** 10.1186/s13065-018-0469-3

**Published:** 2018-09-29

**Authors:** Sraa Abu-Melha

**Affiliations:** Department of Chemistry, Faculty of Science of Girls, King Khaled University, Abha, Saudi Arabia

**Keywords:** *N*-(pyridin-2-yl)hydrazinecarbothioamide, Single-crystal X-ray, Spectral characterization, Molecular docking

## Abstract

*N*-(pyridin-2-yl)hydrazinecarbothioamide has been synthesized and characterized by single-crystal X-ray and spectroscopic techniques. Furthermore, its geometry optimization, calculated vibrational frequencies, non-linear optical properties, electrostatic potential and average local ionization energy properties of molecular surface were being evaluated using Jaguar program in the Schrödinger’s set on the basis of the density functional concept to pretend the molecular geometry and predict properties of molecule performed by the hybrid density functional routine B3LYP. Furthermore, the docking study of *N*-(pyridin-2-yl)hydrazinecarbothioamide were applied against negative *Escherichia coli* bacterial and gram positive *Staphylococcus aureus bacterial* strains by Schrödinger suite program using XP glide protocol.
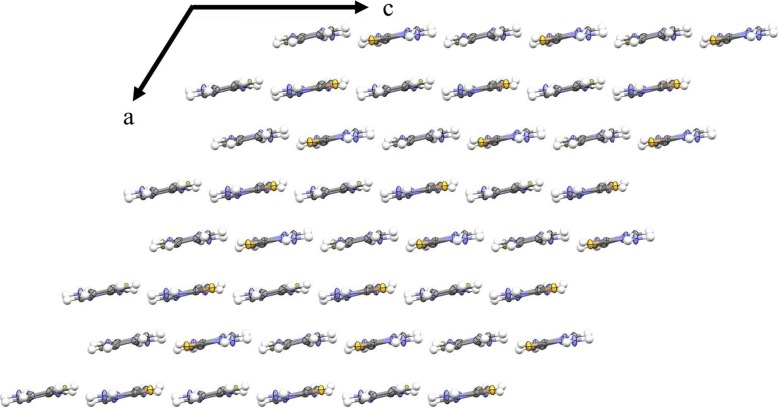

## Introduction

Compounds containing sulfur and nitrogen atoms appear to display antimicrobial activity; antiviral [1, 2], antifungal [3], antibacterial [4, 5], antitumor [6, 7], anticarcinogenic [8–10] and insulin mimetic properties [11]. The antitumor action could be credited to the hindrance of DNA production by the alteration in the reductive transformation of ribonucleotide to deoxyribonucleotide [8]. Thiosemicarbazides have also been utilized for spectrophotometric detection of metals [12–14], gadget applications with respect to media communications and optical storage [15, 16]. Thiosemicarbazides are well-known source in heterocyclic synthesis. They also exist in tautomeric C=S (thione) and (C–S) thiol forms [17]. The presence of tautomeric forms as an equilibrium combination in solution is basic for their adaptable chelating behavior. From these application, we reported the isolation, X-ray crystal characterization, DFT computational studies using B3LYP, molecular interaction docking studies and biological applications of *N*-(pyridin-2-yl)hydrazinecarbothioamide. This study aims to investigate the stability of different isomers either in solid state or solution and show the synergy between the experimental and theoretical data.

## Experimental

### Equipment and materials

All the substances were bought from different high quality sources and used as it is without any additional refining. The infra-red spectrum (4000–400 cm^−1^) by means of KBr discs was measured utilizing a Mattson 5000 FTIR spectrophotometer.

^1^H NMR spectra was measured utilizing a JEOL 500 MHz NMR spectrometer, in (DMSO-d_6_) at 25 °C using TMS as an internal standard. D_2_O solvent is applied to approve the assignment of the NH– and SH– protons. On the other hand, the theoretical calculation of the ^1^H NMR for the different isomers of *N*-(pyridin-2-yl)hydrazinecarbothioamide was done using ACD/SpecManager.

An appropriate crystal for single-crystal X-ray study of the thiosemicarbazide has been selected and mounted onto thin glass fibers. An Enraf–Nonius 590 diffractometer having a Kappa CCD sensor utilizing graphite monochromated Mo-Kα (*λ* = 0.71073 Å) was utilized for collection of the diffraction data of the colorless X-ray single-crystal at normal temperature (25 °C) at the “National Research Center”, Egypt. Reflection data have been recorded in the rotation mode using the φ and ω scan technique with 2*θ*_max_ = 27.49 and 27.45. Without any critical peculiar dissipation, Friedel pairs have been combined. Changes in lit up volume were kept to a base and were considered by the multiscan interframe scaling [18, 19]. The parameters of the unit cell were determined from least-squares refinement with *θ* in the range 0 ≤ *θ* ≤ 30.11 and 3.05 ≤ *θ* ≤ 30.11. The refinement was completed by full-framework slightest squares strategy on the positional and anisotropic temperature parameters of all non-hydrogen atoms on the basis of *F*^2^ by means of CRYSTALS package [20]. The hydrogen atoms were set in figured positions and refined utilizing riding atoms with a typical settled isotropic thermal parameter [21].

### Synthesis of *N*-(pyridin-2-yl)hydrazinecarbothioamide

**Scheme 1:**
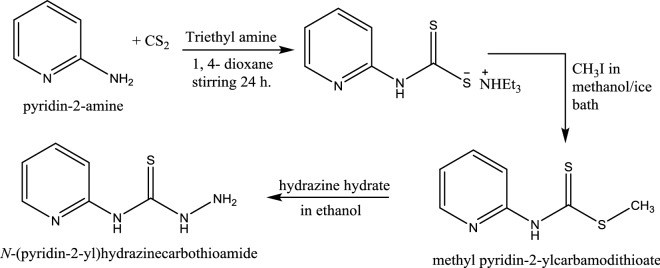
Scheme for synthesis of *N*-(pyridin-2-yl)hydrazinecarbothioamide

*N*-(pyridin-2-yl)hydrazinecarbothioamide is synthesized utilizing Scheme 1. The obtained white precipitate filtered off, splashed using ethanol and desiccated over anhydrous CaCl_2_. (Yield 85%, m.p. 193–195 °C). Crystal suitable for X-ray measurements has been separated by recrystallization from acetonitrile.

### Molecular modeling

Jaguar package [22] in the Schrödinger’s complement [22] was utilized for structural geometry optimization. The density functional principle (DFT) to pretend chemical manners and predict properties of materials performed by the hybrid density functional technique B3LYP [23] implanted with a 6-311G**++basis set.

### Molecular docking

#### Protein preparation

The three-dimensional complex structure of *Escherichia coli* (PDB ID: 1C14) and *Staphylococcus aureus* (PDB ID: 3BL6) were taken from the protein information store [24, 25]. The protein structures were readied utilizing the protein arrangement wizard software in the Schrödinger set [22] in which water molecules (radius > 5Å) and trivial molecules found were expelled from the structure part, disulphide bonds were made and hydrogens were put onto the PDB constructions. Controlled impref minimization having the ordinary inputs was achieved on the structure with improved potentials for fluid reenactments (OPLS-2005) force field. The subsequent structures were utilized for receptor matrix age for docking.

#### Ligand preparation

The investigated compound were equipped utilizing the default procedure of the Ligprep program [22] in the Schrödinger’s set. Glide program [22] in the Schrödinger’s complement was utilized for molecular docking educations. It was docked to the marked protein by means of the glide dock XP practice without any utilization of implement post-docking minimization.

## Result and discussion

### ^1^H NMR of *N*-(pyridin-2-yl)hydrazinecarbothioamide

**Fig. 1:**
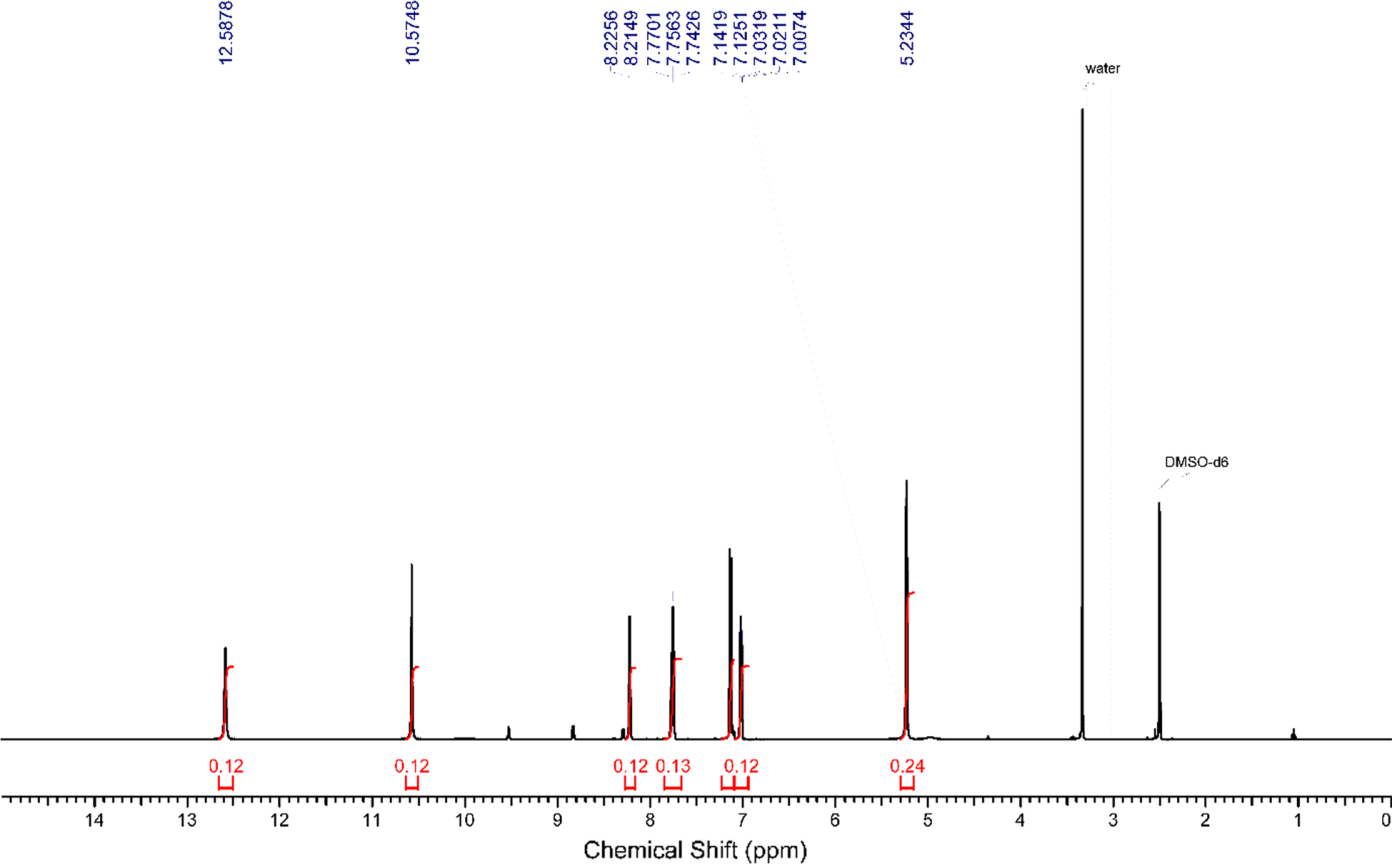
^1^H NMR of N-(pyridin-2-yl)hydrazinecarbothioamide in d_6_-DMSO

**Fig. 2:**
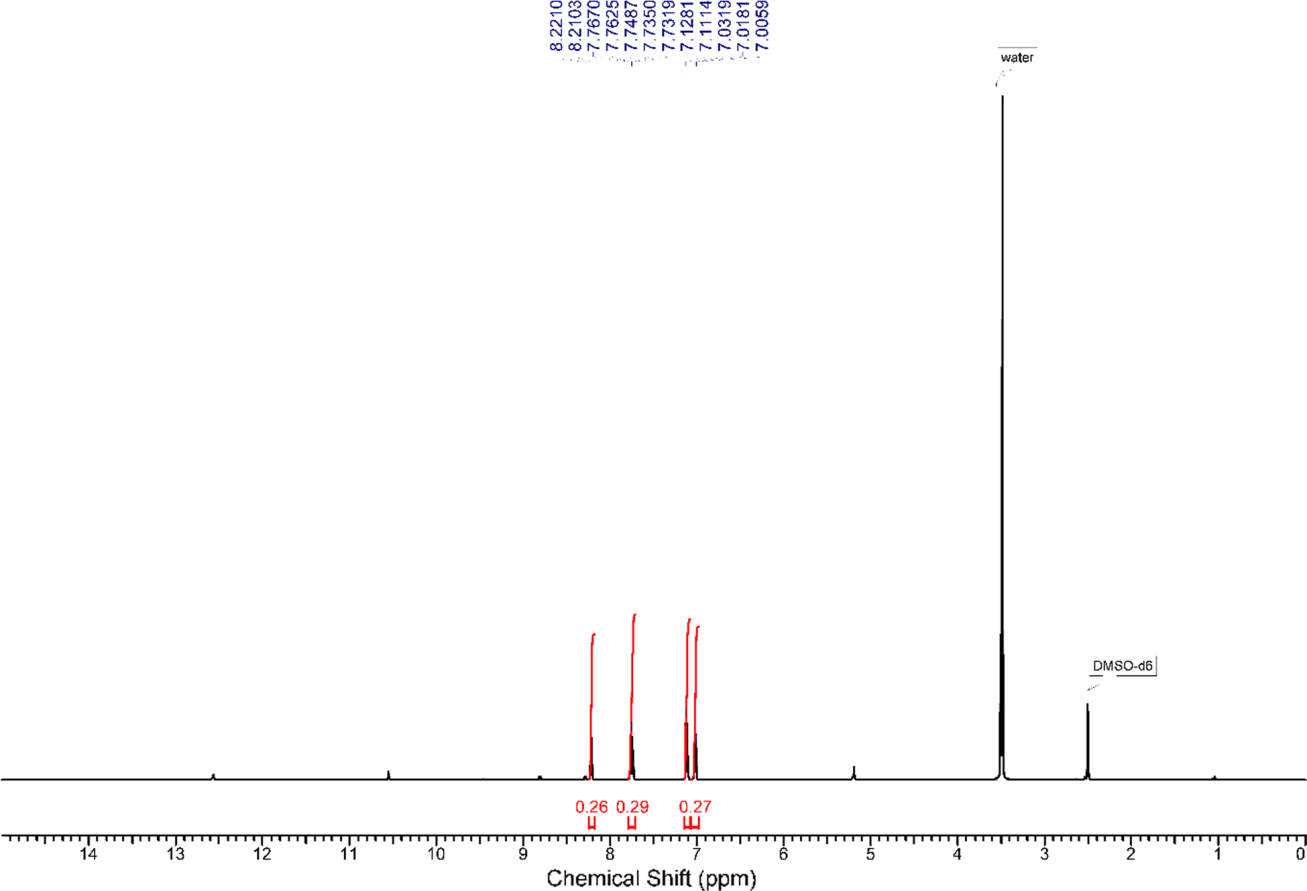
^1^H NMR of *N*-(pyridin-2-yl)hydrazinecarbothioamide in d_6_-DMSO with addition of D_2_O

**Scheme 2:**
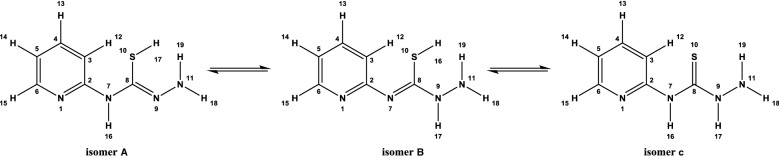
The possible isomers of *N*-(pyridin-2-yl)hydrazinecarbothioamide

**Fig. 3:**
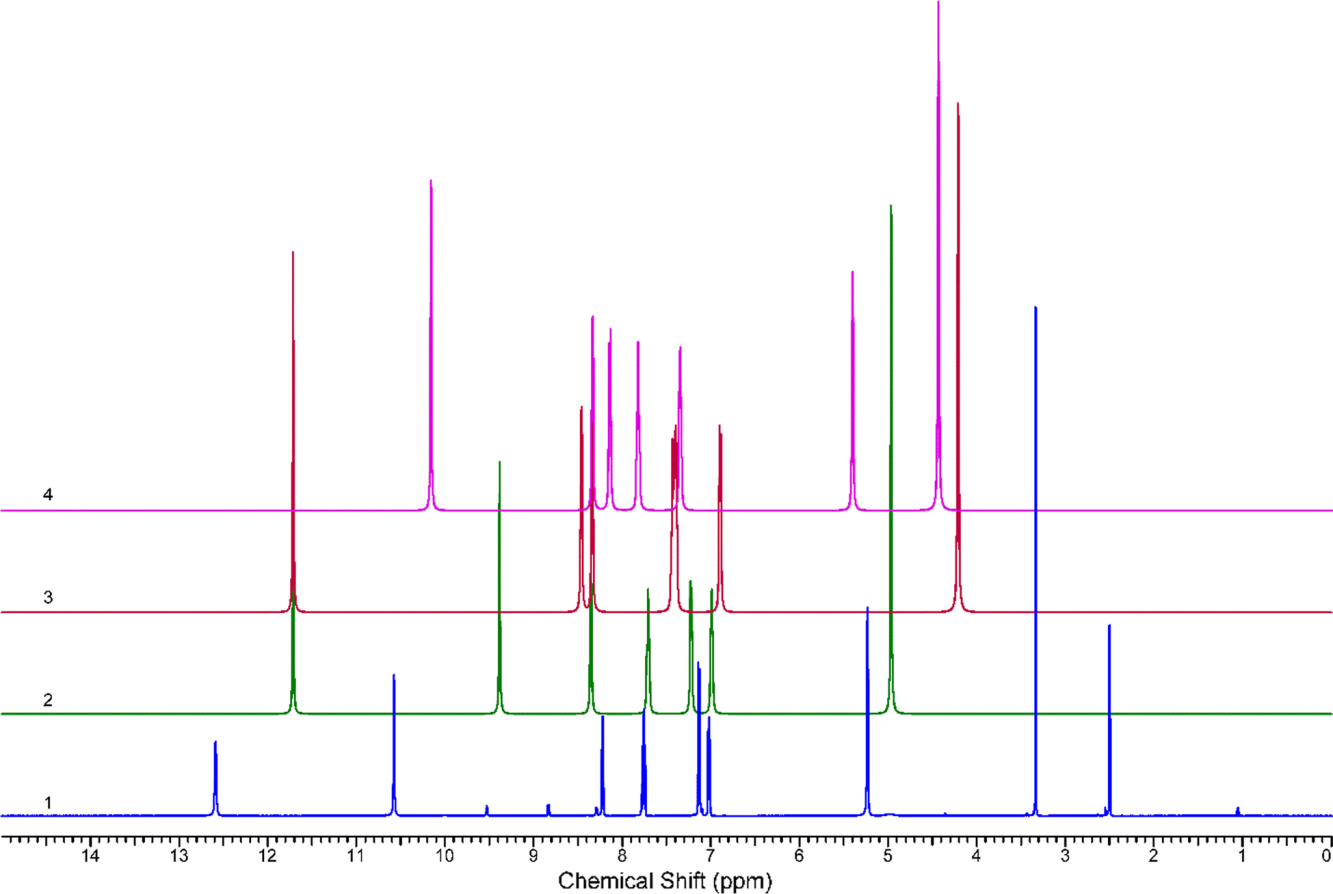
1H NMR of (1) Experimental (2) form (A) (3) form (B) (4) form (C) of *N*-(pyridin-2-yl)hydrazinecarbothioamide

**Fig. 4:**
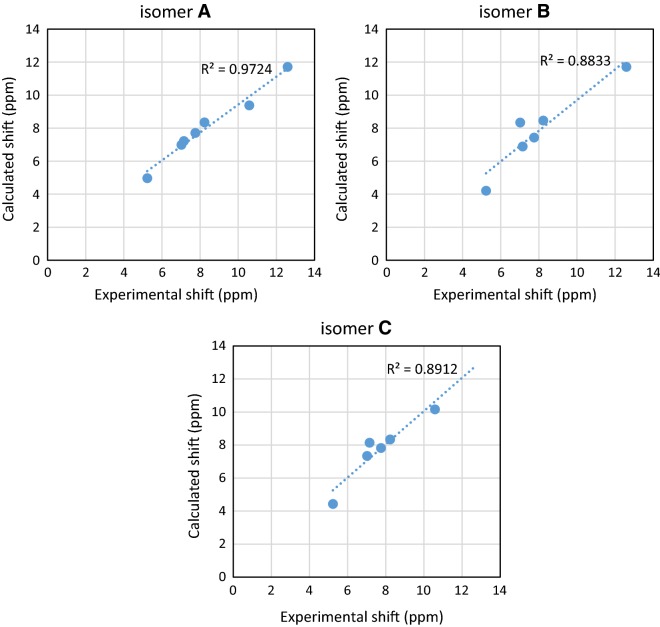
The assignment of linear regression between experimental and calculated shift (ppm) of possible isomers

**Fig. 5:**
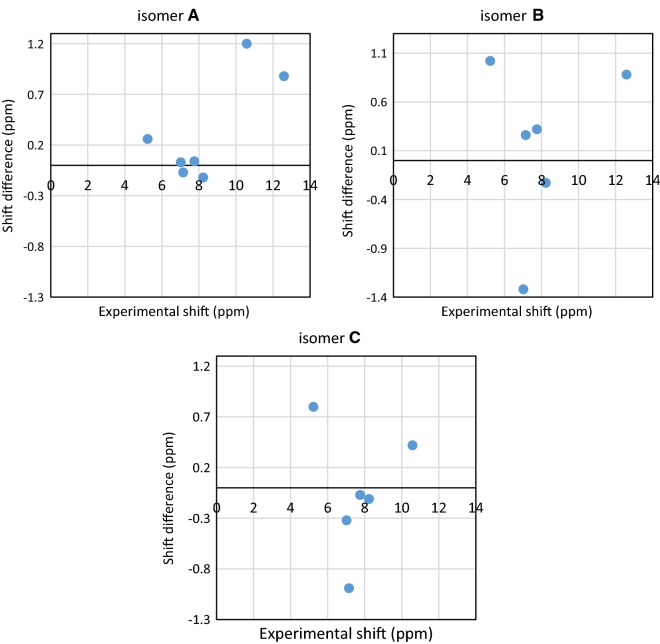
Residual graphs of calculated shift (ppm) of possible isomers related to experimental shift (ppm)

Experimental ^1^H NMR (500 MHz, DMSO-d_6_) ppm 5.23 (br. s., 2 H, [H^18^ and H^19^]) 7.00–7.04 (m, 1 H, H^14^) 7.13 (d, J = 8.41 Hz, 1 H, H^12^) 7.76 (t, J = 6.88 Hz, 1 H, H^13^) 8.22 (d, J = 5.36 Hz, 1 H, H^15^) 10.57 (s, 1 H, H^16^) 12.59 (br. s., 1 H, H^17^) (Fig. 1). The disappearance of the signals of H^16^, H^17^, H^18^ and H^19^ on addition of D_2_O (Fig. 2), which suggests that they are easily exchangeable The presence of a signal at 12.59 ppm attributable to SH proton confirming the presence of the *N*-(pyridin-2-yl)hydrazinecarbothioamide in the thiol form. Additional proof comes from the association of the experimental and theoretical data of the ^1^H NMR for the different isomers of *N*-(pyridin-2-yl)hydrazinecarbothioamide confirmed the presence of the thiosemicarbazide in the thiol form (isomer A) (Scheme 2) in DMSO solution as illustrated in Tables 1 and 2 in addition to Figs. 3, 4 and 5.

**Table 1:** Match factor, RMS of assignment, structure purity, reliability, R^2^ of possible isomers related to experimental ^1^H NMR data

Experimental ^1^H-NMR	Isomer A	Isomer B	Isomer C
Match factor	0.94	0.44	0.19
RMS of assignment (ppm)	0.62	0.87	0.77
Structure purity (%)	99.0	87.0	86.8
Reliability (%)	87.0	74.7	68.5
R^2^	0.97	0.88	0.89

**Table 2:** Comparing of experimental shift (ppm) and calculated shift (ppm) possible isomers

Experimental shift (ppm)	Calculated shift (ppm)		
	Isomer A	Isomer B	Isomer C
5.23	4.97	4.21	4.43
7.02	6.99	8.34	7.34
7.15	7.22	6.89	8.14
7.75	7.71	7.43	7.82
8.23	8.35	8.46	8.34
10.58	9.38	–	10.16
12.59	11.71	11.71	–

### Description of the crystal structure

**Fig. 6:**
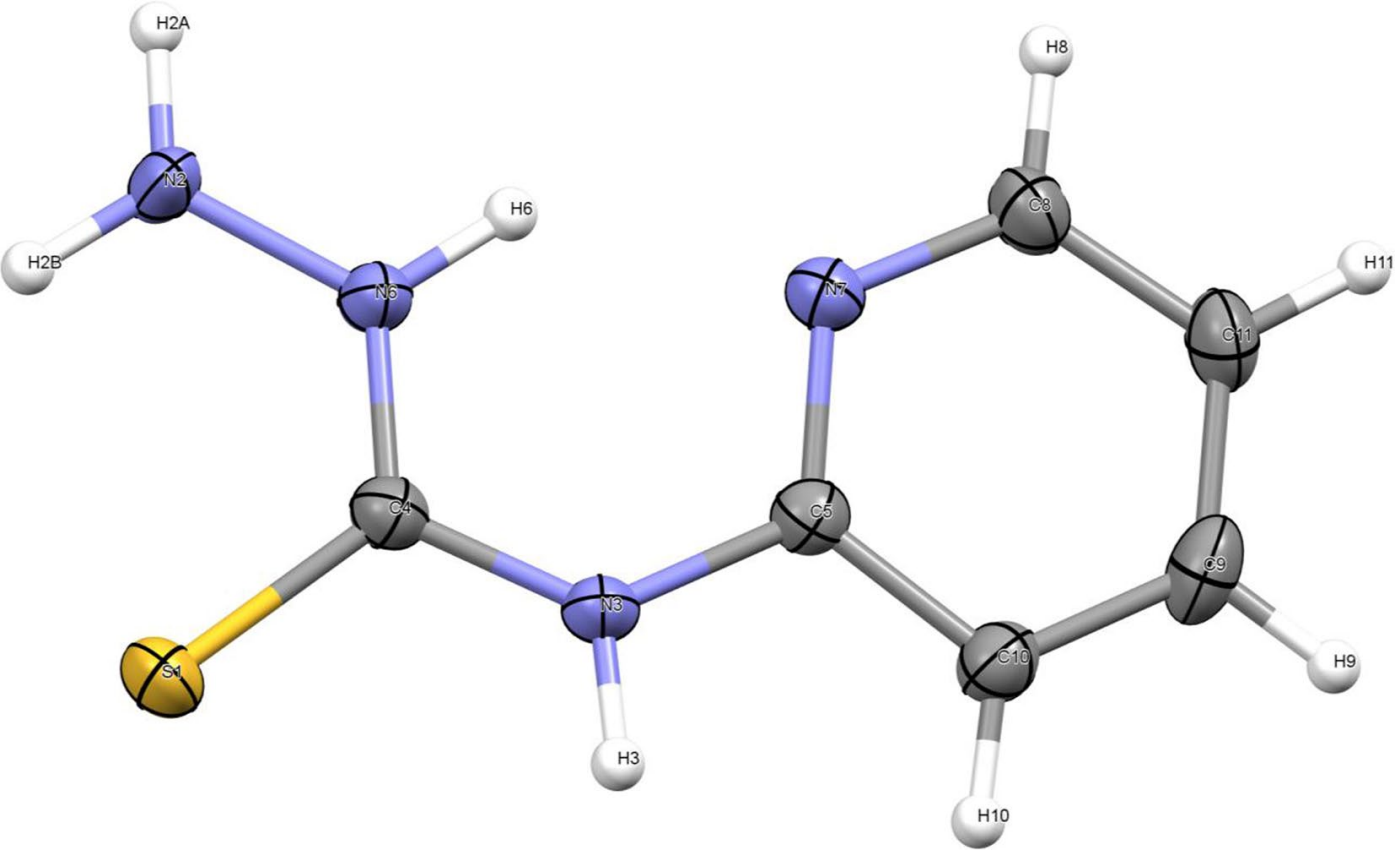
Numbering scheme and atomic displacement ellipsoids drawn at 30% probability level for *N*-(pyridin-2-yl)hydrazinecarbothioamide

The processing data and crystallographic properties of *N*-(pyridin-2-yl)hydrazinecarbothioamide are summarized in Table 3 and Fig. 6 reveals the numbering pattern of *N*-(pyridin-2-yl)hydrazinecarbothioamide thiosemicarbazide. Table 4 illustrate the nominated bond lengths and angles. The ligand crystallizes in the C2/c monoclinic space group with one molecule per asymmetric unit. It comprises of only one independent *N*-(pyridin-2-yl)hydrazinecarbothioamide molecule with no solvent molecules.

**Table 3:** Crystallographic data for *N*-(pyridin-2-yl)hydrazinecarbothioamide

	*N*-(pyridin-2-yl)hydrazinecarbothioamide
Formula	C_6_H_8_N_4_S
Formula weight	168.22
Temperature/K	293
Crystal system	Monoclinic
Space group	*C*2/c
Lattice parameters	
*a*/Å	15.5906 (7)
*b*/Å	10.1719 (5)
*c*/Å	11.1763 (6)
*α/*°	90.00
*β/*°	121.116 (3)
*γ/*°	90.00
*V*/Å^3^	1517.39 (13)
*Z*	8
*D*_calc_/g/cm^3^	1.473
*F*_000_	704
*μ*_Mo-Kα_ Å	0.71073
Reflections collected	7055
Independent reflections	2200
Data/parameters/restrains	2200/100/0
Goodness of fit on *F*^*2*^	1.041
Absorption coefficient mm^−1^	0.36
Final *R* indices (*I *> 2.00*σ*(*I*))	*R*_*1*_ = 0.0602, *wR*_2_ = 0.1649
*R* indices (all data)	*R*_*1*_ = 0.1385, *wR*_2_ = 0.1965
Maximum/minimum residual electron density (e. Å^−3^)	0.397/− 0.489

**Table 4:** Calculated and experimental bond lengths and angles of *N*-(pyridin-2-yl)hydrazinecarbothioamide

Bond length (Å)	Experimental	Calculated	Bond angle (°)	Experimental	Calculated
N(11)–H(19)	0.960	1.017	H(19)–N(11)–H(18)	119.98	106.253
N(11)–H(18)	0.961	1.020	H(19)–N(11)–N(9)	120.95	108.166
N(9)–H(17)	0.959	1.011	H(18)–N(11)–N(9)	119.07	108.041
N(9)–N(11)	0.960	1.403	H(17)–N(9)–N(11)	118.54	113.006
C(8)–S(10)	1.694	1.659	H(17)–N(9)–C(8)	119.25	117.287
C(8)–N(9)	1.322	1.375	N(11)–N(9)–C(8)	121.93	123.165
N(7)–H(16)	0.960	1.011	S(10)–C(8)–N(9)	123.68	123.344
N(7)–C(8)	1.373	1.386	S(10)–C(8)–N(7)	118.41	125.997
C(6)–H(15)	0.960	1.086	N(9)–C(8)–N(7)	117.86	110.598
C(5)–H(14)	0.961	1.083	H(16)–N(7)–C(8)	120.06	115.499
C(5)–C(6)	1.373	1.392	H(16)–N(7)–C(2)	100.36	115.529
C(4)–H(13)	0.960	1.084	C(8)–N(7)–C(2)	129.58	127.311
C(4)–C(5)	1.378	1.393	H(15)–C(6)–C(5)	116.44	120.511
C(3)–H(12)	0.960	1.084	H(15)–C(6)–N(1)	119.79	115.736
C(3)–C(4)	1.368	1.388	C(5)–C(6)–N(1)	123.76	123.746
C(2)–N(7)	1.393	1.411	H(14)–C(5)–C(6)	119.30	120.536
C(2)–C(3)	1.404	1.401	H(14)–C(5)–C(4)	122.56	121.507
N(1)–C(6)	1.339	1.334	C(6)–C(5)–C(4)	118.12	117.957
N(1)–C(2)	1.333	1.329	H(13)–C(4)–C(5)	120.68	120.811
			H(13)–C(4)–C(3)	119.40	120.179
			C(5)–C(4)–C(3)	119.92	119.003
			H(12)–C(3)–C(4)	122.34	121.139
			H(12)–C(3)–C(2)	119.57	120.609
			C(4)–C(3)–C(2)	118.09	118.243
			N(7)–C(2)–C(3)	118.27	118.948
			N(7)–C(2)–N(1)	119.09	117.693
			C(3)–C(2)–N(1)	122.65	123.293
			C(6)–N(1)–C(2)	117.45	117.728

The least-squares planes as defined by the carbon atoms of the phenyl group besides the nitrogen atom of the pyridine ring and the atom directly bonded to it on the one hand and the carbon and nitrogen atoms of the chain-type substituent on the other hand enclose an angle of 9.51°. The C=S bond length is found at 1.694 Å which is intermediate between the usual values for a S(?)-C(*sp*^2^) single (1.75–1.78 Å) and a double (1.59 Å) bond and in good agreement with other reported thioketones [26]. The two C(=S)–N bonds differ slightly in length with values of 1.322 Å and 1.373 Å with the longer bond established towards the nitrogenous atom bonded to the aromatic system. The N–N bond is measured at 1.417 Å corresponds to a single bond. The most striking evidence for the single bond character of the N(11)–N(9) bond is that the hydrogen atoms, placed in the positions calculated on the assumption that N(7) is trigonally hybridized in the mean molecular plane, lead to H… H contact, with an adjacent molecule, which are greatly smaller (1.22 Å) than the value of the van der Waals radii (2.40 Å). In the crystal, intra- and intermolecular classical hydrogen bonds of the N–H–N type are apparent next to C–H–S contacts whose range falls below the sum of van-der-Waals radii (2.40 Å) of the atoms participating in the construction stability [27]. The two molecules can be assumed to be practically coplanar and to be joined together in a dimer by the hydrogen bonds with the neighboring molecule.

**Fig. 7:**
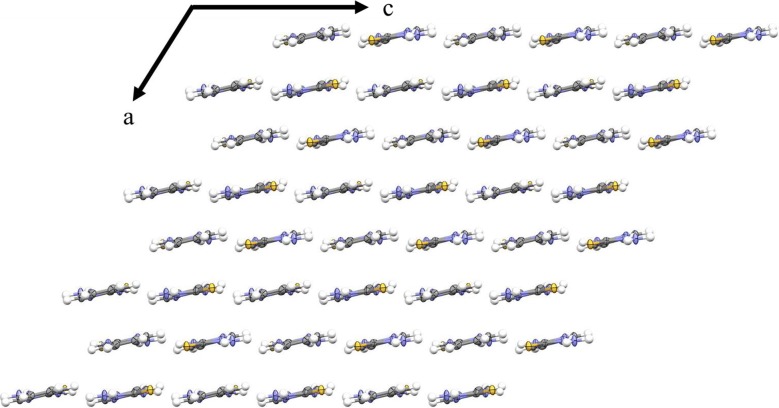
Packing diagram of *N*-(pyridin-2-yl)hydrazinecarbothioamide showing molecular stacking along the ac-plane

**Fig. 8:**
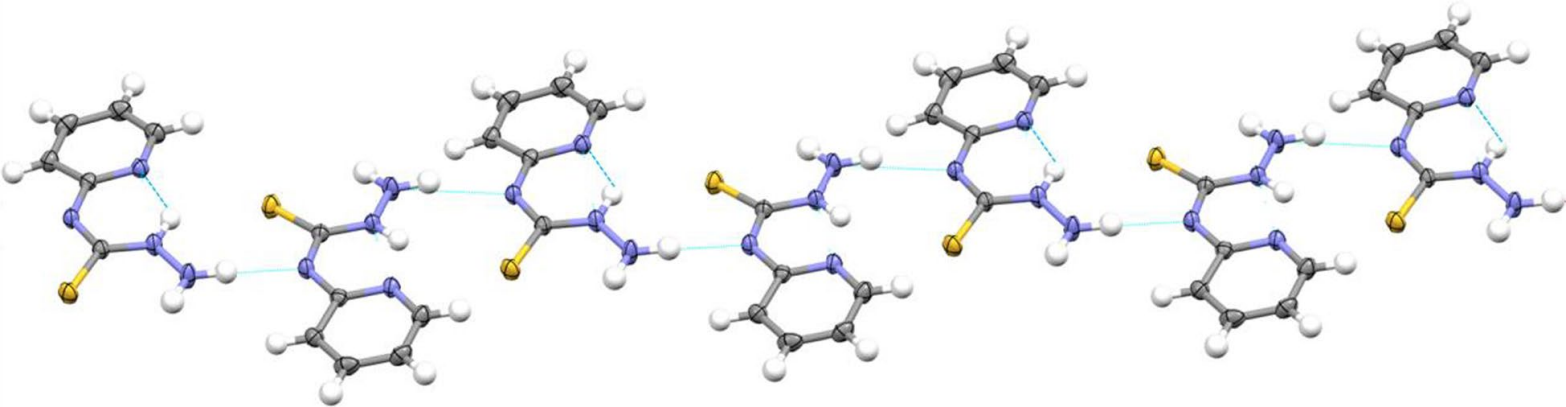
Hydrogen bridges (green lines) along the ac plane of the unit cell

As an issue of guideline, the packing figure of *N*-(pyridin-2-yl)hydrazinecarbothioamide construction (Fig. 7) is very straightforward. It consists of layers of ligand molecules with the same orientation (all the molecule pointing in the same direction), which are held together via hydrogen bonds as appeared in Fig. 8. There are *π*–*π* stacking interactions with distances about 3.348–3.46 Å between the molecules of each row, prompting heaps of stacked ligand molecules. The pyridine rings of the adjacent ligand molecule are not coplanar in the solid state, which is probably due to stacking effects. In the crystal packing, offset *π*–*π* stacking interactions have been observed between neighboring pyridine rings of two molecules in the head-to-tail arrangement forming similar dimeric packing structures, as displayed in Fig. 8. The centroid–centroid separations between the dimeric pairs are 3.586 Å.

### Molecular computational calculation

#### Geometry optimization using DFT

Structure 1 illustrates the optimized structure and numbering scheme of *N*-(pyridin-2-yl)hydrazinecarbothioamide. From the analysis of the estimated and measured data for the bond lengths and angles Table 4 one can observe the similarity between the estimated and measured data.

**Structure 1:**
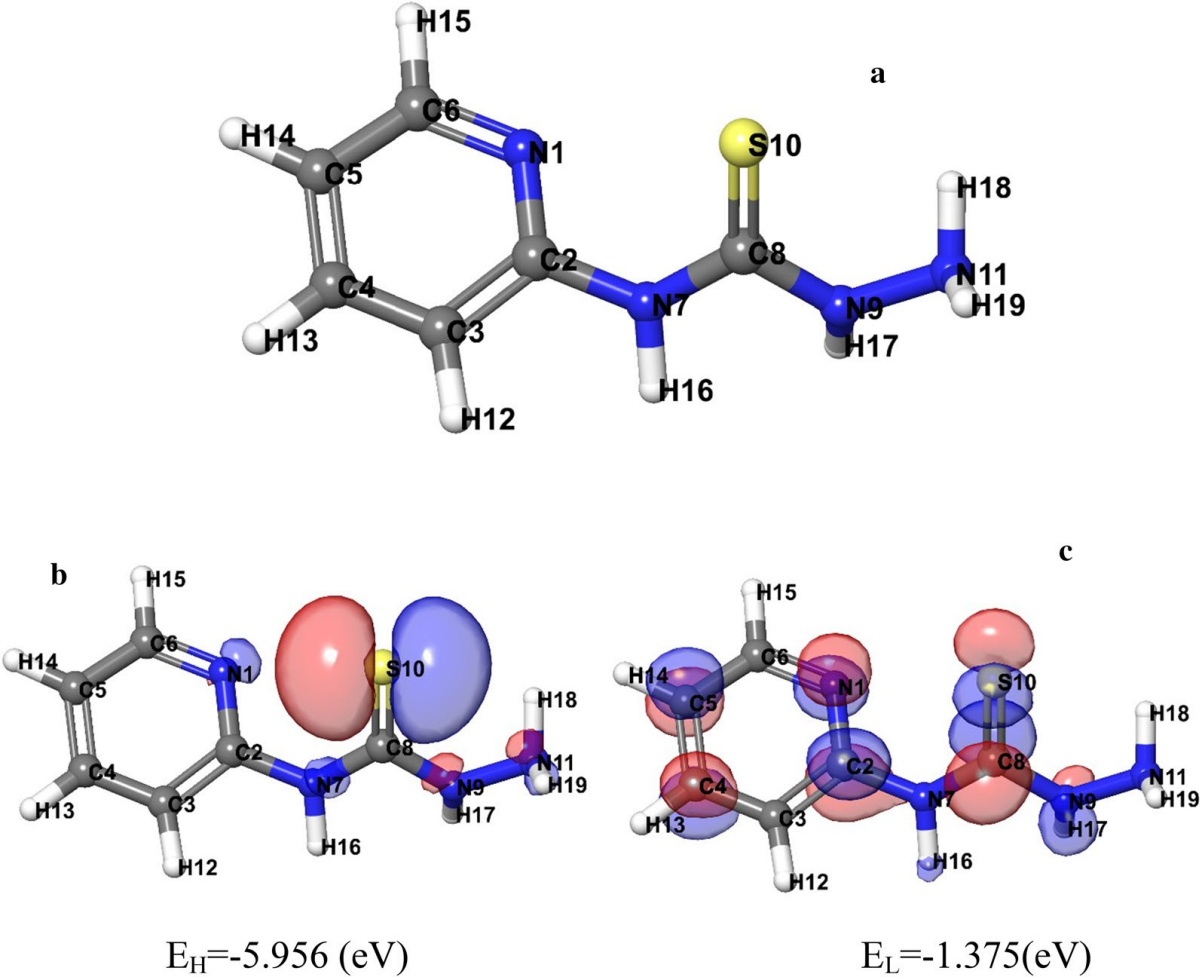
Geometry optimization using DFT method of ligands **a** *N*-(pyridin-2-yl)hydrazinecarbothioamide, **b** HOMO and **c** LUMO

The calculated energy components and energies of both HOMO (π donor) and LUMO (π acceptor) Table 5 are main parameters in quantum chemical studies. Where, HOMO is the orbital that behaves as an electron giver, LUMO is the orbital that behave as the electron acceptor and these molecular orbitals are known as the frontier molecular orbitals (FMOs) Structure 1.

**Table 5:** Calculated energy components, E_HOMO_, E_LUMO_, energy band gap (*E*_*H*_−* E*_*L*_), chemical potential (μ), electronegativity (χ), global hardness (η), global softness (S) and global electrophilicity index (ω) for *N*-(pyridin-2-yl)hydrazinecarbothioamide

Energy components	Kcal/mol	Energetic parameters	
Nuclear repulsion	4.14 × 10^5^	*E*_H_ (eV)	− 5.956
Total one-electron terms	− 1.55 × 10^6^	*E*_*L*_ (eV)	− 1.375
Electron-nuclear	− 2.08 × 10^6^	(*E*_H_ − *E*_L_) (eV)	− 4.580
Kinetic	5.32 × 10^5^	*Χ* (eV)	3.665
Total two-electron terms	6.04 × 10^5^	µ (eV)	− 3.665
Coulomb	6.60 × 10^5^	*η* (eV)	2.290
Exchange and correlation	− 5.58 × 10^4^	*S* (eV^−1^)	1.145
Electronic energy	− 9.48 × 10^5^	*ω* (eV)	2.933
Gas phase energy	− 5.34 × 10^5^	Ϭ (eV)	0.436

DFT technique illustrates the discernment of the molecular arrangements and expects the chemical reactivity. The energies of gas stage, FMOs (*E*_HOMO_, *E*_LUMO_), electronegativity (*χ*), energy band gap that clarifies the inevitable charge exchange communication inside the particle inside the molecule, global hardness (*η*), chemical potential (*µ*), global electrophilicity index (*ω*) and global softness (*S*) [28, 29] are recorded in Table 5.

In numerous responses, the overlap amongst HOMO and LUMO orbitals assumed as an administering reason, where in compounds under examination; the orbitals with the higher molecular orbital coefficients can be considered as the fundamental destinations of the complexation. The energy gap (*E*_HOMO _− *E*_LUMO_) is a noteworthy stability index simplify the description of both kinetic stability and chemical reactivity of the investigated moieties [30]. The energy gap of ligand is small showing that charge transfers easily in it and this influences the biological activity of the molecule, which agree with experimental data of antibacterial, and antifungal activities. Furthermore, the small quantity of energy difference can be assigned to the groups that enter into conjugation [31].

#### Experimental IR and vibrational calculation

**Fig. 9:**
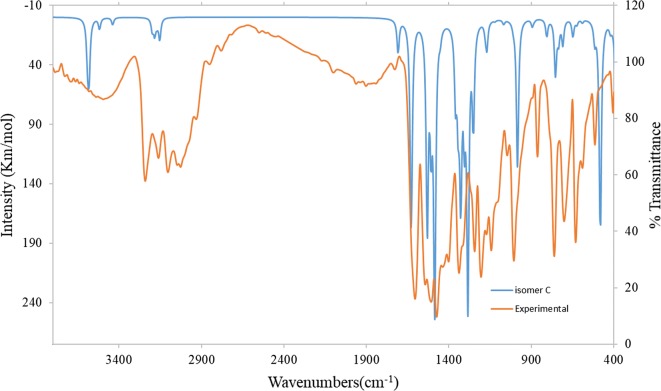
Comparison of experimental and theoretical IR spectra of *N*-(pyridin-2-yl)hydrazinecarbothioamide

**Fig. 10:**
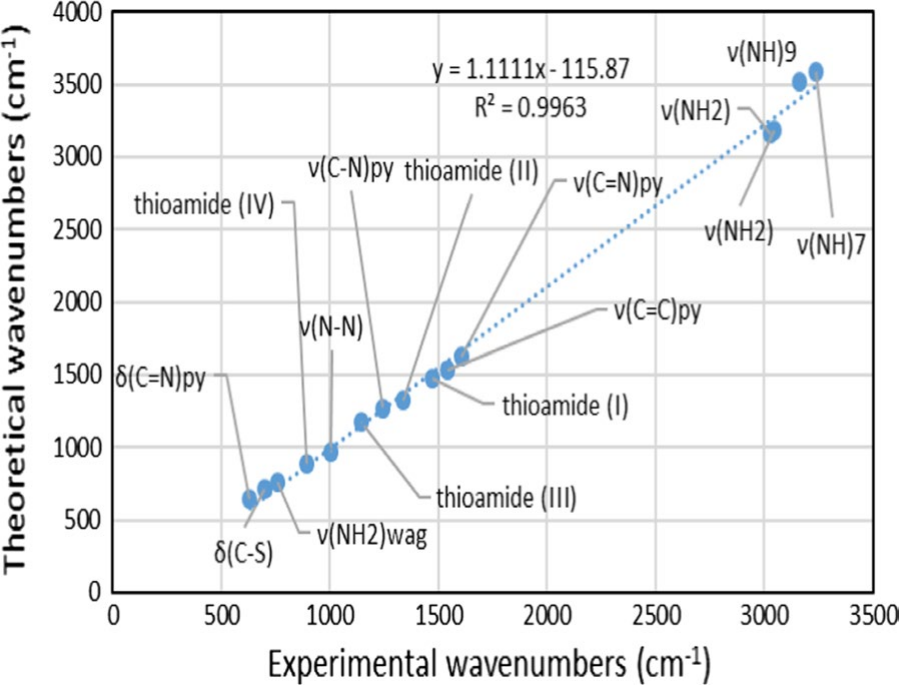
The linear regression between the experimental and theoretical frequencies of *N*-(pyridin-2-yl)hydrazinecarbothioamide

In order to get the spectroscopic signature of ligands compounds, a frequency calculation analysis were carried out. The calculations were completed for free molecule in vacuum, while experiments were performed for solid sample (Table 6), so there are small differences between hypothetical and measured vibrational frequencies as illustrated in Fig. 9. The modes of vibrations are very complex because of the low symmetry of ligands. Particularly, in plane, out of plane and torsion vibrations have the greatest difficultly to allocate because of the involvement with the ring vibrations and with the substituent vibrations. However, there are some strong frequencies useful to characterize in the IR graph. The relationship that showed the similarities among the calculated and measured data is illustrated in Fig. 10 which confirm the existence of the *N*-(pyridin-2-yl)hydrazinecarbothioamide in the thione form (isomer C). The relations between the calculated and experimental wavenumbers are linear for ligand and described by ν_cal_ = 1.1111 ν_Exp_− 115.87 with R^2^ = 0.9963.

**Table 6:** Expermintal and theoretical wavenumber (cm^−1^) of *N*-(pyridin-2-yl)hydrazinecarbothioamide

Function group	Experimental wavenumber (cm^−1^)	Theoretical wavenumber (cm^−1^)
ν(NH_2_)	3025, 3046	3155, 3185
ν(NH)^7^	3241	3589
ν(NH)^9^	3160	3515
ν(C=N)_py_	1606	1631
ν(C=C)_py_	1544	1530
ν(C–N)_py_	1243	1270
δ(C=N)_py_	632	650
ν(NH_2_)_wag_	761	755
ν(N–N)	1006	971
Thioamide (I)	1474	1475
Thioamide (II)	1337	1330
Thioamide (III)	1143	1175
Thioamide (IV)	893	890
δ(C–S)	701	710

As a glance of table and figures, one can conclude the following remarks:

i. The linear regression between the experimental and theoretical frequencies confirms the existence of the *N*-(pyridin-2-yl)hydrazinecarbothioamide in the thione form (isomer C).
ii. The relations between the hypothetical and measured data is linear and described by equation ν_cal_ = 1.1111 ν_Exp_ − 115.87 with R^2^ = 0.9963.
iii. The two bands at 3240 and 3160 cm^−1^ were attributed to the stretching (NH)^7^ and (NH)^9^ groups, respectively [32].
iv. The bands observed at 1606, 1544 and 1243 cm^−1^ assigned to ν(C=N), (C=C) and (C–N) stretching of pyridine rings, respectively [33]. Also the out of plane and in plane binding frequencies of (C=N)_py_ appeared at 632 [34].
v. The thiosemicarbazide exhibited ν(–NH_2_ → =NH) at 3025 and 3046 cm^−1^. While ν(–NH_2_) wagging appeared at 761 cm^−1^.
vi. A band at 1006 cm^−1^ corresponding to ν(N–N) [35].
vii. The thioamide group (HN–C=S) displayed four thioamide bands (I–IV) at 1474 cm^−1^ (I), 1337 cm^−1^ (II), 1143 cm^−1^ (III) and 893 cm^−1^ (IV) [36–39].

#### Non-linear optical (NLO) properties

The quantum chemistry based prediction of NLO possessions of *N*-(pyridin-2-yl)hydrazinecarbothioamide has an essential part for the design of materials in communication technology, signal processing and optical interconnections [40]. The total static dipole moment μ, the average linear polarizability $\overline{\alpha }$, the anisotropy of the polarizability ∆α, and the first hyper-polarizability β can be calculated as reported by Sajan et al. [40]. Table 7 illustrates the ingredients of dipole moment, polarizability and the average first hyper-polarizability of *N*-(pyridin-2-yl)hydrazinecarbothioamide framework.

**Table 7:** Calculated dipole moments (D), polarizability and the first hyperpolarizability components (a.u.) for ligand compounds

Dipole moment (a.u.)		First hyperpolarizability (a.u.)	
μ_x_	− 0.25195	β_xxx_	− 3.91 × 10^2^
μ_y_	1.55977	β_yyy_	− 74.10
μ_z_	0.75273	β_zzz_	− 36.80
μ	1.75013	β_xyy_	1.03 × 10^2^
Polarizability (a.u.)		β_xzz_	42.60
α_xx_	168.596	β_yxx_	− 10.60
α_xy_	− 16.288	β_yzz_	49.30
α_xz_	− 7.067	β_zxx_	− 33.00
α_yy_	122.347	β_zyy_	31.70
α_yz_	13.479	β_xyz_	38.00
α_zz_	93.355	Σβ_x_	− 2.46 × 10^2^
$\overline{\upalpha}$	128.099	Σβ_y_	− 35.40
∆α	299.3229	Σβ_z_	− 38.10
		β	251.4374

The estimated data were changed into Debye Å^3^ and electrostatic units (e.s.u.) utilizing the well-known conversion relations (for μ: 1 a.u. = 2.5416 Debye; for α: 1 a.u. = 0.14818 Å^3^; for β: 1 a.u. = 8.641 × 10^−33^ e.s.u.) [41]. Urea is utilizes as an acute parameter for comparison studies because it has a decent NLO activity (μ = 1.3732 Debye, $\overline{\alpha }$ = 3.8312 Å^3^ and β = 3.7289 × 10^−31^ cm^5^/e.s.u.). Furthermore *N*-(pyridin-2-yl)hydrazinecarbothioamide have parameters μ = 4.4481 Debye, $\overline{\alpha }$ = 18.9817 Å^3^, ∆α = 44.3551 Å^3^, and β = 2.1727 × 10^−30^ cm^5^/e.s.u.

The first hyper-polarizability of *N*-(pyridin-2-yl)hydrazinecarbothioamide is greater than that of urea 5.82 times, respectively. According to the magnitude of β, the *N*-(pyridin-2-yl)hydrazinecarbothioamide under study may be have a potential applicant in the improvement of NLO materials due to they have a worthy non-linear property.

#### Electrostatic potential (ESP) and average local ionization energy (ALIE) properties on molecular surface

Electrostatic potential *V*(*r*) and average local ionization energy $\overline{I}$(*r*) of molecule have confirmed to be active guides to its reactive behavior [42].

Electrostatic potential V(r) and average local ionization energy $\overline{\text{I}}$(r) of all frameworks were shown in Structures 2 and 3, respectively. Also, estimated molecular surface data showed in Table 8. This table include the following parameters:

**Structure 2:**
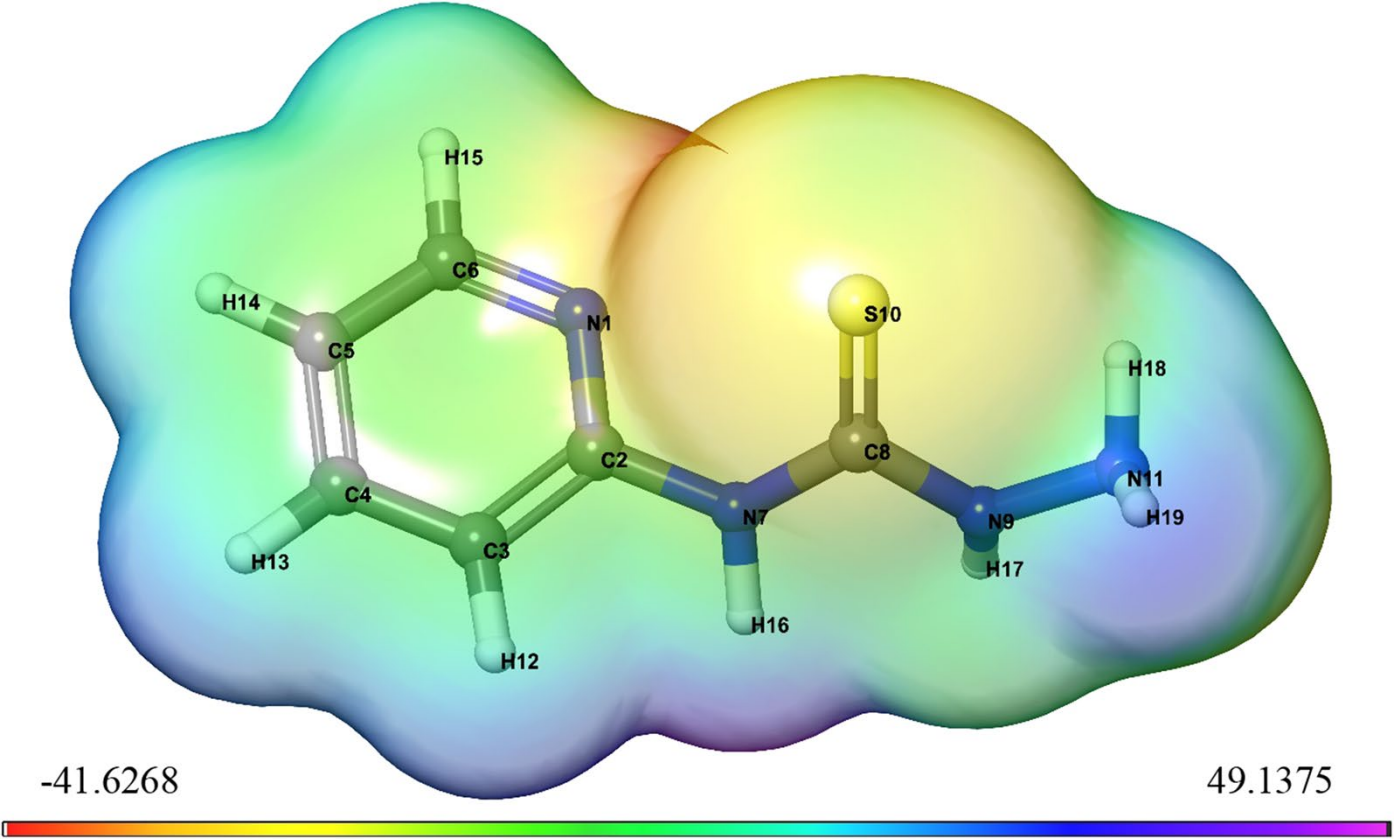
Surface structure of ESP using DFT method for *N*-(pyridin-2-yl)hydrazinecarbothioamide

**Structure 3:**
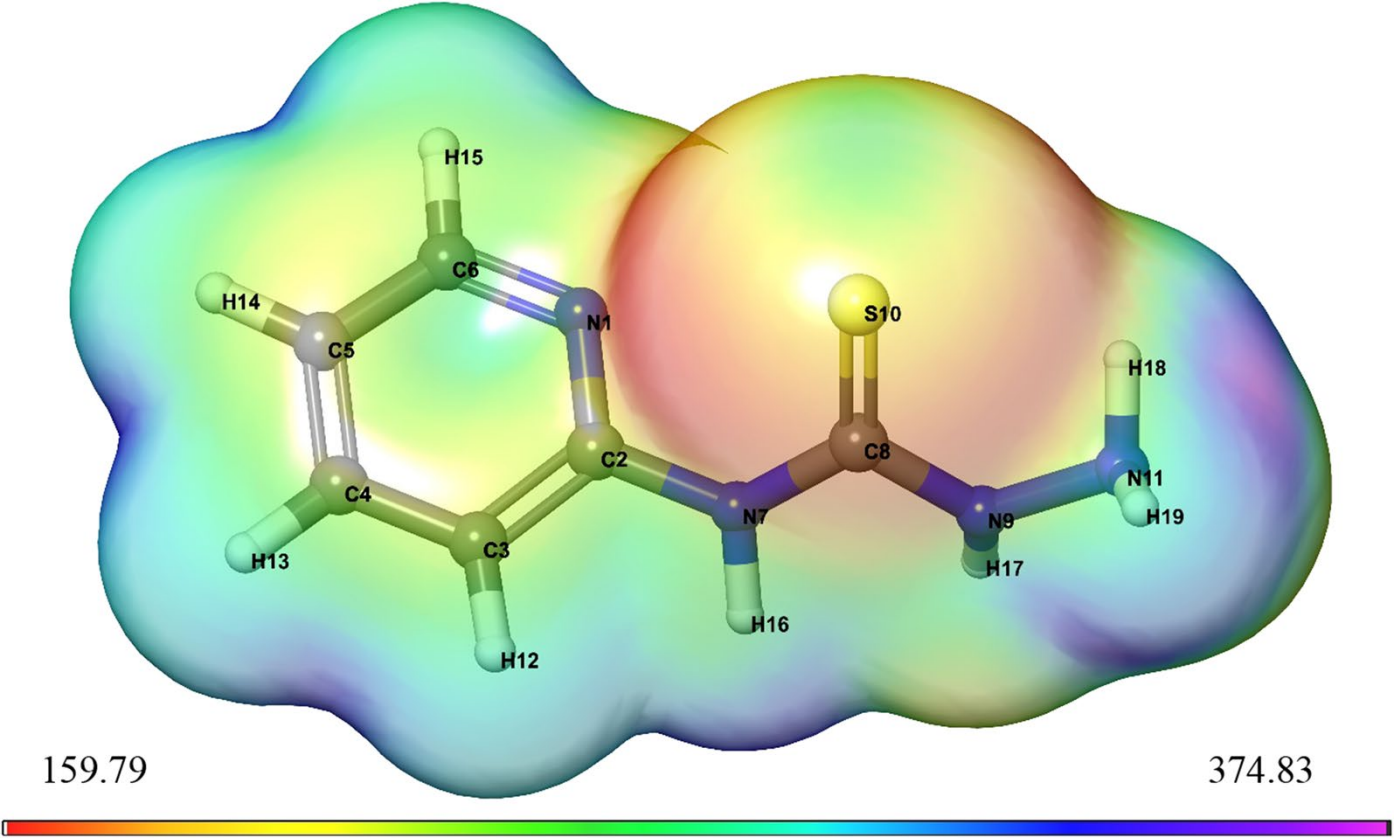
Surface structure of ALIE using DFT method for *N*-(pyridin-2-yl)hydrazinecarbothioamide

**Table 8:** Computed molecular surface properties (ESP) and (ALIE) of compound

*V*_*s,min*_	− 41.62	$\sigma_{ + }^{2}$	116.01	$\overline{I}$_*S, min*_	159.79
*V*_*s,max*_	48.98	$\sigma_{ - }^{2}$	106.73	$\overline{I}$_*S, max*_	374.80
$\overline{V}$_*S*_	1.10	$\sigma_{tot}^{2}$	222.74	$\overline{I}$	252.47
$\overline{V}_{s}^{ + }$	15.03	ν	0.25	$\overline{I}$_*S, ave*_	39.74
$\overline{V}_{s}^{ - }$	− 15.96	Π	15.37		

Units: *V*_*s,min*_, *V*_*s,max*_, $\overline{V}$_*S*_, $\overline{V}_{s}^{ + }$, $\overline{V}_{s}^{ - }$, Π, $\overline{I}$_*S, min*_, $\overline{I}$_*S, max*_, $\overline{I}$_*S*_ and $\overline{I}$_*S, ave*_ are in kcal/mol; $\sigma_{ + }^{2}$, $\sigma_{ - }^{2}$, $\sigma_{tot}^{2}$ are in (kcal/mol)^2^; ν is unitless

i. The data of the most positive and most negative *V*_*S,max*_ and *V*_*S,min*_.
ii. Overall surface potential value $\overline{V}$_*S*_, its positive and negative averages $\overline{V}_{s}^{ + }$ and $\overline{V}_{s}^{ - }$.
iii. The internal charge transfer (local polarity) Π, which is deduced as a meter for the internal charge separation and it is present even in molecules with zero dipole moment because of the symmetry.
iv. The variances, $\sigma_{ + }^{2}$, $\sigma_{ - }^{2}$ and $\sigma_{tot}^{2}$ which reflect the strengths and variabilities of the positive, negative and overall surface potentials [43].
v. An electrostatic balance parameter ν = 0.25, that illustrate the extent of the equilibrium amongst the positive and negative potentials; when $\sigma_{ + }^{2} = \sigma_{ - }^{2}$
vi. The most positive and most negative $\overline{I}$_*S,max*_ and $\overline{I}$_*S,min*_ and the average over the surface of the local ionization energy $\overline{I}$_*S,ave*_.

From Table 8 we notice that *N*-(pyridin-2-yl)hydrazinecarbothioamide has the internal charge separation, Π = 15.37 kcal mol^−1^, may be due to it was structurally quite symmetric.

In Structures 2 and 3 is displayed the *V*_*S*_*(r)* and $\overline{I}$_*S*_*(r)* on surfaces of *N*-(pyridin-2-yl)hydrazinecarbothioamide. These structures show the locations of the various most positive and most negative *V*_*S*_*(r)*, *V*_*S,max*_ and *V*_*S,min*_, and the highest and lowest $\overline{I}$_*S*_*(r)*, $\overline{I}$_*S,max*_ and $\overline{I}$_*S,min*_. There are often several local maxima and minima of each property on a studied molecular surface. The most negative electrostatic potential on *N*-(pyridin-2-yl)hydrazinecarbothioamide surface is related to the nitrogen (N1) of pyridine ring, *V*_*S,min*_ = − 41.62 kcal mol^−1^, followed by weaker value − 38.6 kcal mol^−1^ on the sulfur (S10). Thus, *V*_*S*_*(r)* would wrongly predict electrophilic attack to occur preferentially at the nitrogen. In contrast, the lowest values of $\overline{I}$_*S*_*(r)* placed on the (S10), with $\overline{I}$_*S,min*_ = 159.79 kcal mol^−1^; also, there is an $\overline{I}$_*S,min*_ by the hydrogen (H18), but it is much higher, 165.39 kcal mol^−1^. Thus, $\overline{I}$_*S*_*(r)* shows the most reactive, least-tightly-bound electrons to be at the (S10), properly indicating these sites to be most susceptible to electrophiles. On the other hand, the very strongly positive electrostatic potential of the hydrogen (H16), *V*_*S,max*_ = 48.98 kcal mol^−1^, and the *V*_*S,min*_ = − 41.62 kcal mol^−1^ of the nitrogen (N1) indicate their tendencies for noncovalent hydrogen bonding, as a donor and an acceptor, respectively.

### Molecular docking

The molecular interaction of *N*-(pyridin-2-yl)hydrazinecarbothioamide for inhibition against *E. coli* and *S. aureus* are represented in Structures 4, 5, 6 and 7 shows exchanges with the active site residues with dock score − 4.523 and − 5.265 kcal/mol for both *E. coli* and *S. aureus*, respectively. The affinity of *N*-(pyridin-2-yl)hydrazinecarbothioamide against *E. coli* is resulting from two hydrogen bonds interaction (NH_2_ → TYR156 and (NH)^7^ → ALA196). While, the interaction with *S. aureus* resulting from the molecular hydrogen bonds interaction (NH_2_ → SER75 and (NH)^9^ → H_2_O → GLU11).

**Structure 4:**
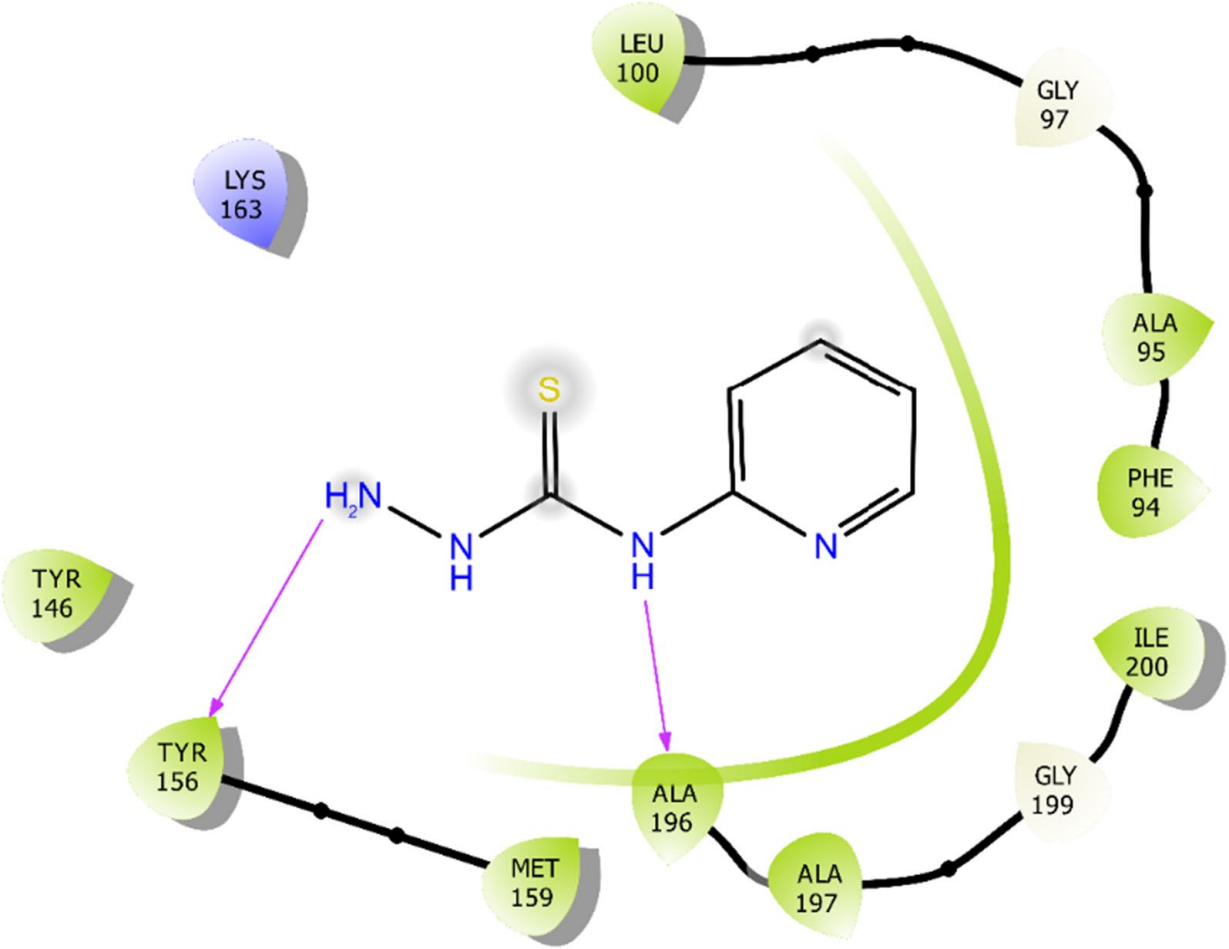
2D molecular interaction of *N*-(pyridin-2-yl)hydrazinecarbothioamide for inhibitor to *E. coli*

**Structure 5:**
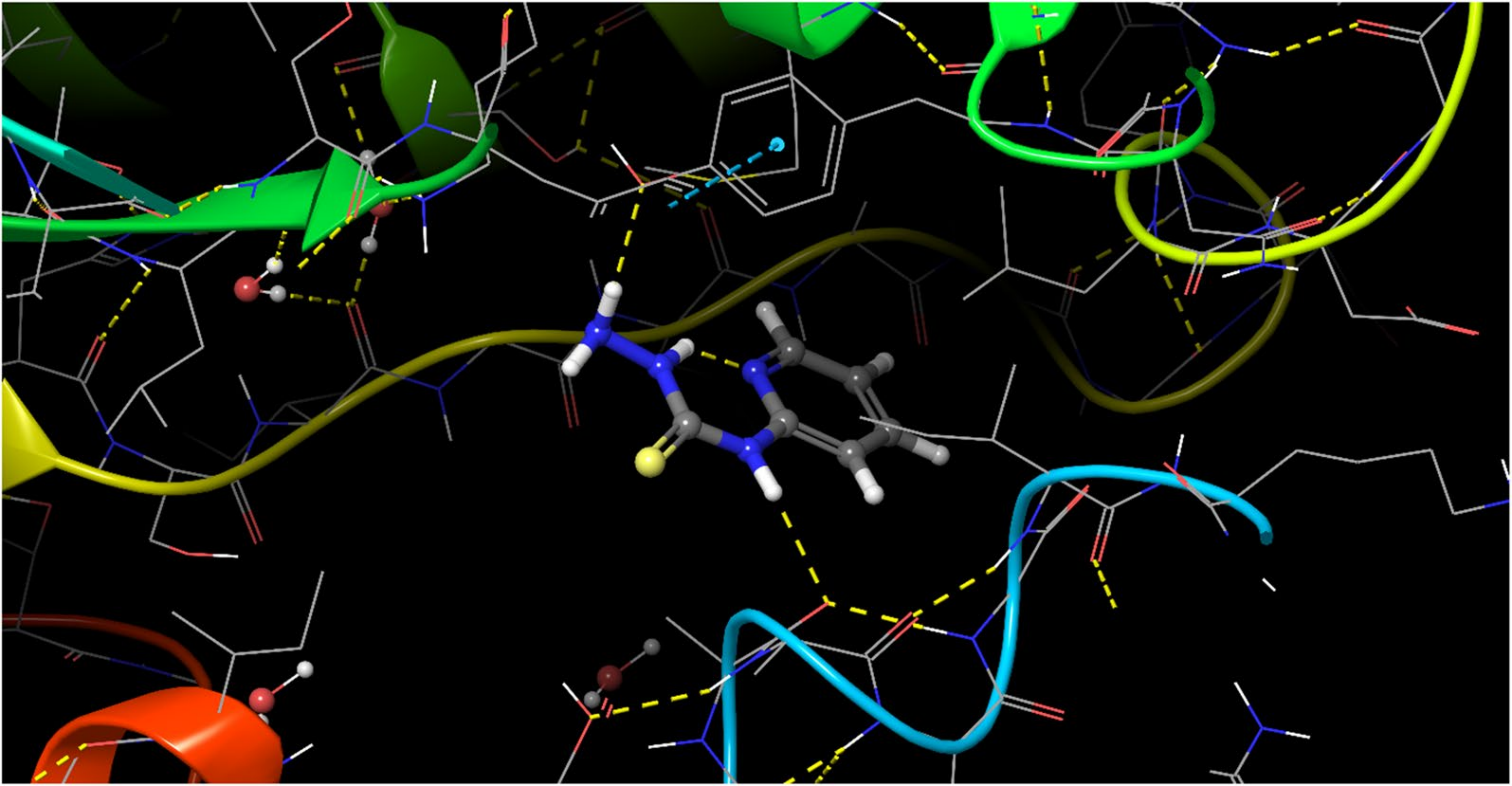
3D molecular interaction of *N*-(pyridin-2-yl)hydrazinecarbothioamide for inhibitor to *E. coli*

**Structure 6:**
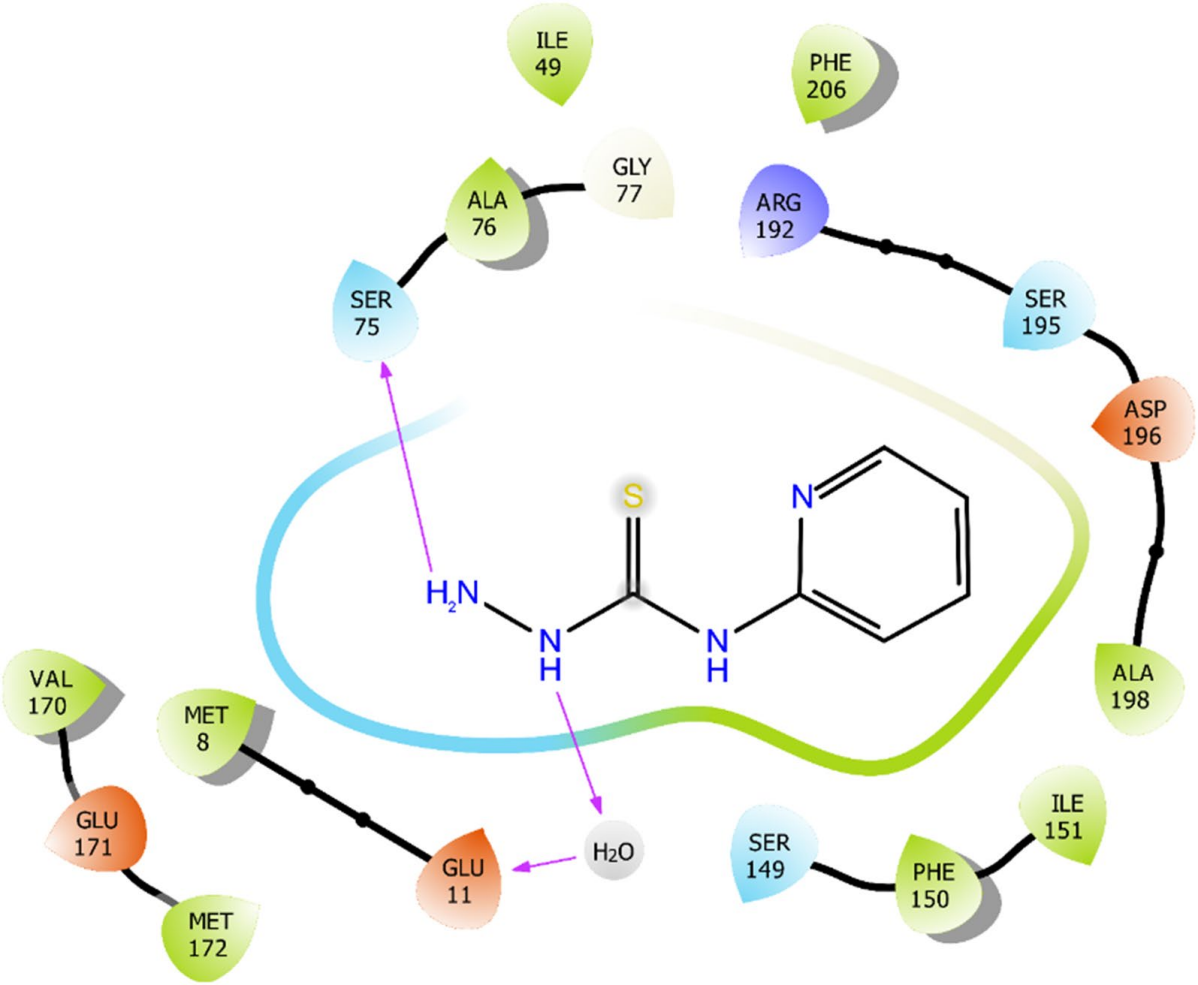
2D molecular interaction of *N*-(pyridin-2-yl)hydrazinecarbothioamide for inhibitor to *S. aureus*

**Structure 7:**
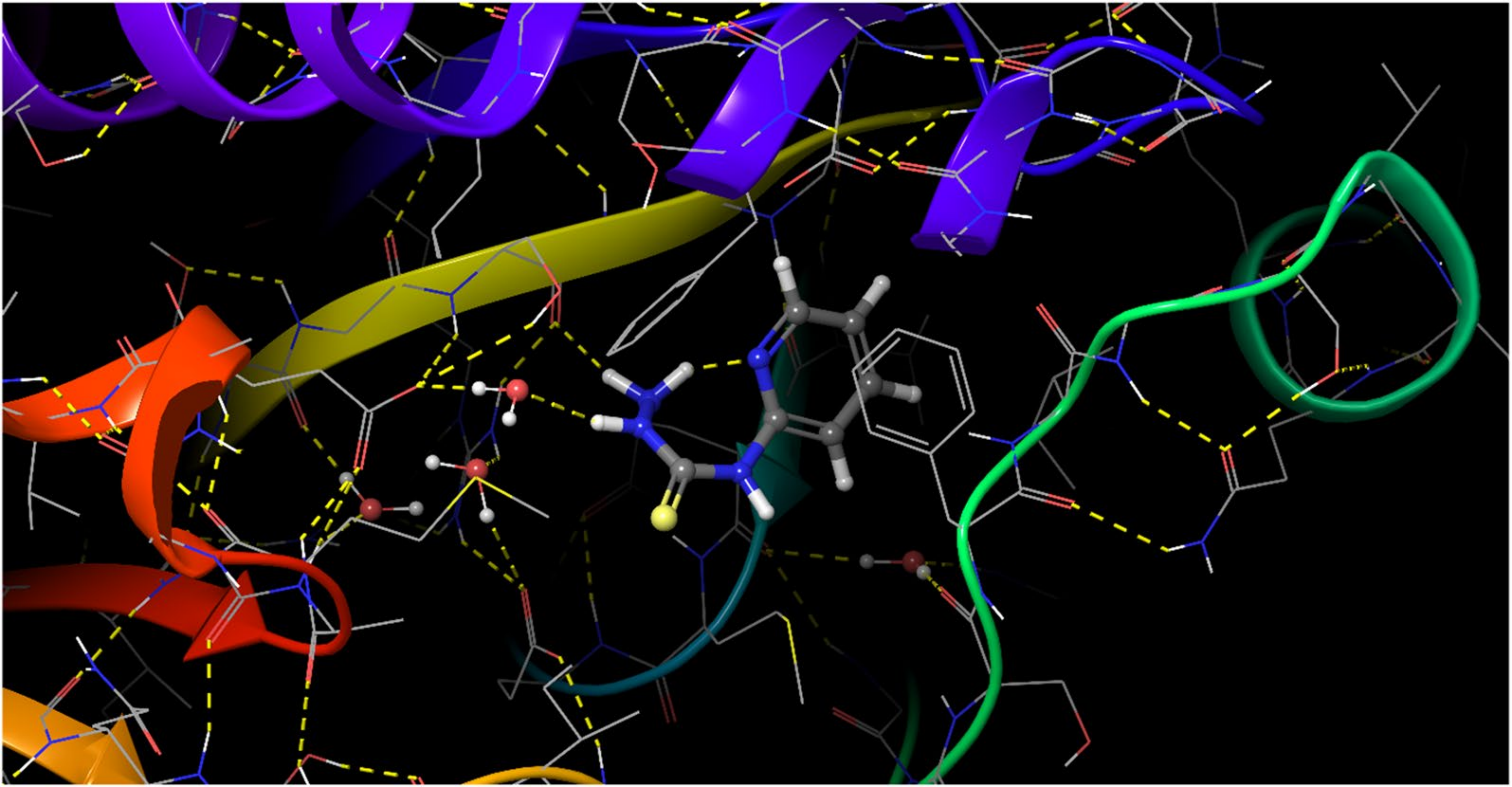
3D molecular interaction of *N*-(pyridin-2-yl)hydrazinecarbothioamide for inhibitor to *S. aureus*

## Conclusion

Novel thiosemicarbazide; *N*-(pyridin-2-yl)hydrazinecarbothioamide has been isolated and described utilizing single-crystal X-ray and ^1^HNMR. Additionally, its geometry optimization, calculated vibrational frequencies, non-linear optical properties, electrostatic potential and average local ionization energy properties of molecular surface were being assessed by means of Jaguar program in the Schrödinger’s set on the basis of the density functional concept (DFT) to pretend the molecular geometry and predict properties of molecule performed by the hybrid density functional routine B3LYP. Finally, the docking study of *N*-(pyridin-2-yl)hydrazinecarbothioamide were applied against negative *E. coli* bacterial and gram positive *S. aureus bacterial* strains by Schrödinger suite program using XP glide protocol.
